# DivCom: A Tool for Systematic Partition of Groups of Microbial Profiles Into Intrinsic Subclusters and Distance-Based Subgroup Comparisons

**DOI:** 10.3389/fbinf.2022.864382

**Published:** 2022-05-12

**Authors:** Evangelia Intze, Ilias Lagkouvardos

**Affiliations:** ^1^ School of Science and Technology, Hellenic Open University, Patras, Greece; ^2^ Core Facility Microbiome, ZIEL – Institute for Food and Health, Technical University Munich, Freising, Germany; ^3^ Institute of Marine Biology, Biotechnology and Aquaculture, Hellenic Centre for Marine Research, Heraklion, Greece

**Keywords:** microbial profiles, beta diversity, *de novo* clustering, reference distance, PAM

## Abstract

When analyzing microbiome data, one of the main objectives is to effectively compare the microbial profiles of samples belonging to different groups. Beta diversity measures the level of similarity among samples, usually in the form of dissimilarity matrices. The use of suitable statistical tests in conjunction with those matrices typically provides us with all the necessary information to evaluate the overall similarity of groups of microbial communities. However, in some cases, this approach can lead us to deceptive conclusions, mainly due to the uneven dispersions of the groups and the existence of unique or unexpected substructures in the dataset. To address these issues, we developed divide and compare (DivCom), an automated tool for advanced beta diversity analysis. DivCom reveals the inner structure of groups by dividing their samples into the appropriate number of clusters and then compares the distances of every profile to the centers of these clusters. This information can be used for determining the existing interrelation of the groups. The proposed methodology and the developed tool were assessed by comparing the response of anemic patients with or without inflammatory bowel disease to different iron replacement therapies. DivCom generated results that revealed the inner structure of the dataset, evaluated the relationship among the clusters, and assessed the effect of the treatments. The DivCom tool is freely available at: https://github.com/Lagkouvardos/DivCom.

## 1 Introduction

Over the last 20 years, the field of microbiome research has been experiencing exponential growth, mainly powered by advances in sequencing technology. A significant amount of this body of research has been focused on how dysbiotic microbial communities are linked with pathological conditions (Coker et al., 2018; Harbison et al., 2019; Harbison et al., 2019; Hufnagl et al., 2020). In addition, the importance of microbes has been recognized in other fields spanning from agricultural and biotechnological applications to ecological and environmental interventions (Lian et al., 2018; Qiu et al., 2019).

Nowadays, several pipelines, tools, and platforms are dedicated to analyzing microbiome datasets. Specialized tools include R-based packages such as vegan (Oksanen et al., 2015), phyloseq (McMurdie and Holmes, 2013), and SIAMCAT (Wirbel et al., 2021). Pipelines, like QIIME2 (Bolyen et al., 2019), mothur (Schloss et al., 2009), and Rhea (Lagkouvardos et al., 2017), usually offer streamlined analytical functionalities with minimal programming requirements. However, more tools and methodologies are under development to reflect the growth in our understanding of the topic and accommodate our needs for more specialized analytics.

Beta diversity, the measure of diversity between two samples, is one of the most widely used concepts in microbiome data analysis (Lin et al., 2015; Wagner et al., 2018). Beta diversity does not focus on the abundance of specific bacterial taxa but takes into account the overall microbial community structure. The usage of an appropriate metric function results in a single measurement (distance) of similarity or dissimilarity that can be used to examine the relations among the samples in a study. Metrics like Bray-Curtis (Bray and Curtis, 1957), weighted or unweighted Unifrac (Lozupone et al., 2011), and Jaccard distance (Jaccard, 1912) are commonly used for exploratory and ordination analyses. In a limited number of studies, the quantification of beta diversity measures has been utilized to gain better insights into the community dynamics (Halfvarson et al., 2017; Suzuki et al., 2020).

Clustering a group without using labels or prior knowledge of the data is defined as unsupervised clustering. Unsupervised clustering does not use any external information and relies only on the pairwise distances of the samples. Since this type of clustering shares similar principles with the *de novo* OTU picking (Navas-Molina et al., 2013), here in this study, we will borrow this term, and we will call the process of the unsupervised clustering as “*de novo* clustering” of the microbial profiles. This procedure can be extremely helpful for revealing substructures of a dataset that are unknown or have not been predicted during the study design (Ramette, 2007). The proposed concept of the enterotypes (Arumugam et al., 2011) is one of the most known cases where *de novo* clustering revealed intrinsic substructures in the human gut microbiota. Also, *de novo* clustering contributed significantly to drawing conclusions in the studies of Paetzold et al. (2019) and later García-Mantrana et al. (2020), which investigated skin and maternal microbiomes, respectively. Although both beta diversity and *de novo* clustering techniques are commonly used by individual researchers, no standardized procedure, pipeline, or tool integrates and automates their combined use for group comparisons.

Comparing the microbial profile of two or more groups against each other or exploring the relationship between control and intervention groups is part of a typical workflow for many studies (Morris et al., 2013; Prast-Nielsen et al., 2019; Ventura et al. 2019). Through this process, the dissimilarity between the members of each group can be used to determine the level of differentiation among the examined groups.

The problem that arises is that the approaches used to analyze the microbial datasets can lead us, in some cases, to wrong assumptions or incomplete conclusions. Among others, there are three main obstacles in the currently applied methodologies: the first is derived by the dimensionality reduction process (Calle, 2019), the second by the statistical tests (Xia and Sun, 2017), and the third by not taking into consideration the unique substructure of the data (Gupta et al., 2017; He et al., 2018). Because of the dimensionality reduction process and the selected distance metric, there is a high chance of producing a distorted image of the data (Calle, 2019; Hawinkel et al., 2019). Relying only on the visual representation of the ordination plots can lead us to misleading conclusions about the existing relationship among the profiles of the different groups. The suggested practice for evaluating groups’ dissimilarities is through the application of a multivariable statistical test (Knight et al., 2018) like PERMANOVA (permutational multivariate analysis of variance) (Anderson, 2001), PERMDISP (permutational analysis of multivariate dispersions) (Anderson, 2006), or ANOSIM (analysis of similarities) (Clarke, 1993). However, even this practice can also produce inaccurate or deceptive outcomes mainly due to the lack of homogeneity between the groups, the different levels of their dispersion (Anderson et al., 2008; Warton et al., 2012), and the wrong use and interpretation of the results of the statistical tests.

**FIGURE 1:**
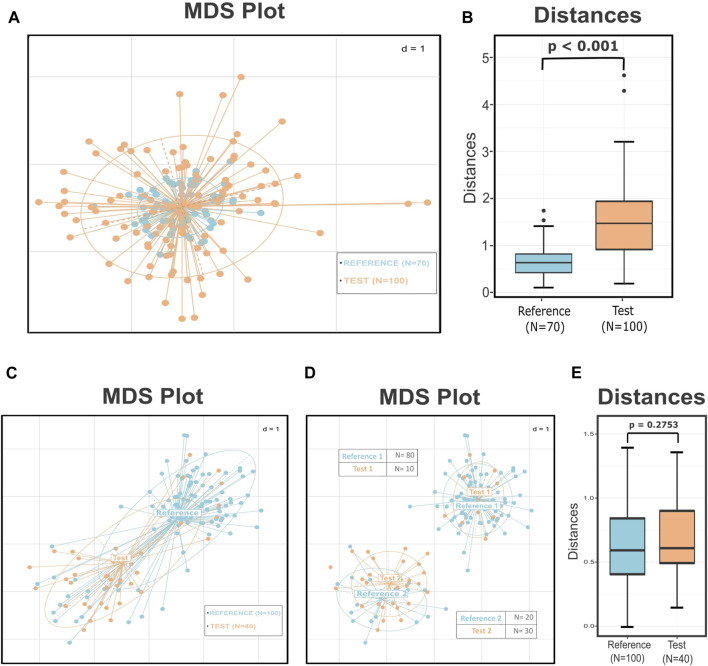
Simulated data demonstrating how different dispersion among groups influences the results of the statistical tests and how the substructures within the groups and the uneven sampling of these subclusters can lead to a misleading interpretation of the data. **(A)** MDS plot presenting two groups with the same center but according to the PERMDISP test significantly different variances (*p* < 0.001). The *p*-value of the PERMANOVA and ANOSIM tests for these groups is 0.838 and 0.556, respectively, leading to not rejecting the null hypothesis that the two groups are drawn from the same distribution. **(B)** Boxplots presenting the distances of the test points from the center of the reference group. The *p*-value of the Wilcoxon rank sum test is less than 0.001, implying correctly that these two groups are significantly different. **(C)** MDS plot that presents two groups with similar dispersions (*p* = 0.35) but seemingly different centers. The results of the PERMANOVA (*p* < 0.001) and ANOSIM (*p* < 0.001) tests indicate that these two groups are well-separated. **(D)** MDS plot illustrating the subgroups derived by the *de novo* clustering of the two groups. The dataset now consists of two pairs of highly related clusters with different representations in the two subgroups. **(E)** Boxplots presenting the distribution of the distances of every test sample from their closest reference center. According to the *p*-value of the Wilcoxon rank sum test (*p* = 0.2753), the points of the two groups do not differ significantly in their values. The perceived difference comes from the unequal representation of the two subgroups in the final dataset.

To illustrate these issues, we present two hypothetical cases in which wrong conclusions can easily be drawn if we follow the widely applied microbiome data analysis practices. In the first case, we simulated two groups that have the same center and a similar number of samples but significantly different dispersions (Figure 1A). The visual inspection of the plot suggests structural differences among these two groups; however, the PERMANOVA (*p* = 0.838) and ANOSIM (*p* = 0.556) tests affected by the same centers and the different dispersions (PERMDISP: *p* < 0.001) returned a high probability that both groups originate from the same distribution. Although PERMANOVA and ANOSIM tests are fairly robust methods, they have their own limitations and are sensitive to different dispersions. Relying only on these results can obscure the information about the substantially different structures of the dataset.

In the next case, according to PERMDISP, the two groups have similar dispersions (*p* = 0.35) but appear to have different centers (Figure 1C); this observation is also supported by the results of the PERMANOVA (*p* < 0.001) and ANOSIM tests (*p* < 0.001). These facts could lead the researcher to conclude that samples originating from group “Test” have significantly different profiles than those of group “Reference.” The PERMANOVA test assumes that there is only one distribution from which every group is sampling. However, *de novo* clustering of both groups reveals that each of them consists of two well-defined subgroups (Figure 1D). The use of statistical tests like PERMANOVA on groups composed of multiple subgroups can lead to misleading conclusions. In our instance, the two pairs of subclusters have similar centers and dispersions but different sampling sizes and also uneven number of samples belonging to each subgroup. This unequal representation of the two otherwise related compositions, from which both groups were composed, was the reason for the distorted and misinformative initial image. Thus, revealing the internal structure of the groups could provide us with additional information about the dataset and assist us in preventing errors like those mentioned earlier.

In these two cases, we summarized some of the existing problems in the microbiome beta diversity analysis that sometimes are difficult to be detected and overpassed. The problem presented in the first case is the improper use of the statistical tests or the wrong interpretation of their results. In the first instance, the PERMDISP test provided a clear view that the groups have significantly different dispersions; this should have been a hindrance to applying multivariate tests like PERANOVA or ANOSIM as their results could have been inaccurate. However, in many cases, the power of these tests is overestimated by the researchers, leading them to wrong conclusions. In the second instance, even though we did not have any misuse of the statistical tests, the inner structure of the groups was a key factor in having incorrect results. Unfortunately, in these cases, there is not an easy and reliable alternative for the user to follow: either the researcher will have to rely on the results of the statistical tests with the fear of drawing the wrong conclusions or should alter the exploratory approach of the study.

Herein, we introduce DivCom, a new approach that can be used as an answer to the challenges mentioned earlier. This approach aims to compare different groups in a more efficient and detailed way, and reveal their relations. The central notion behind DivCom is that groups of microbial profiles should not be treated as entireties because valuable information about their unique structural characteristics could be lost. DivCom employs the idea of dividing the groups using *de novo* clustering and then comparing these clusters using beta diversity measures as metrics. According to the methodology of DivCom, the samples of the control group are clustered, and then, the most representative point (centroid/medoid) for each of these clusters is selected. Consequently, all the distances of the remaining test samples from these preselected points are calculated and then assessed.

Applying the DivCom methodology to the previously mentioned simulated cases of Figure 1, we can infer that in the first instance, the distinct structure of the dataset was revealed by comparing the distances of the samples of the two groups from the center (*p* < 0.001) (Figure 1B). Also, in the second case, the distance of the samples from their closest reference center showed that there is no significant difference between the two pairs of groups (Figure 1E). Therefore, even though the commonly used techniques failed to uncover the true relationship of the data, DivCom achieved this by using a distance- and structure-based approach. Also, the use of the centroids reduced the required calculations and comparisons, and produced results that are analogous with those we would have obtained if we had compared all test samples with all the samples of the closest reference cluster (Supplementary Figure S1).

The effectiveness of the method was also evaluated using publicly available gut microbiome data from the study of Lee et al. (2017). The selected research aimed to compare the effect of iron replacement in anemic patients who suffered from inflammatory bowel disease against a group of non-inflamed anemic individuals. All the subjects were randomly separated into two groups, and they followed two different treatments of iron replacement for 3 months. Simply by applying the DivCom approach, we were able to reproduce some of the main findings of the original analysis and also reveal some additional aspects of the data that were unnoticed in the original work. DivCom provides us with a better insight into the data and can be used complementary to the currently applied data analysis pipelines. The proposed methodology is implemented as an automated, open-source, user-friendly, and easily-editable R-based program. The DivCom tool has minimal input requirements, produces several detailed outputs, and is available at: https://github.com/Lagkouvardos/DivCom.

## 2 Materials and Methods

### 2.1 Overview

**FIGURE 2:**
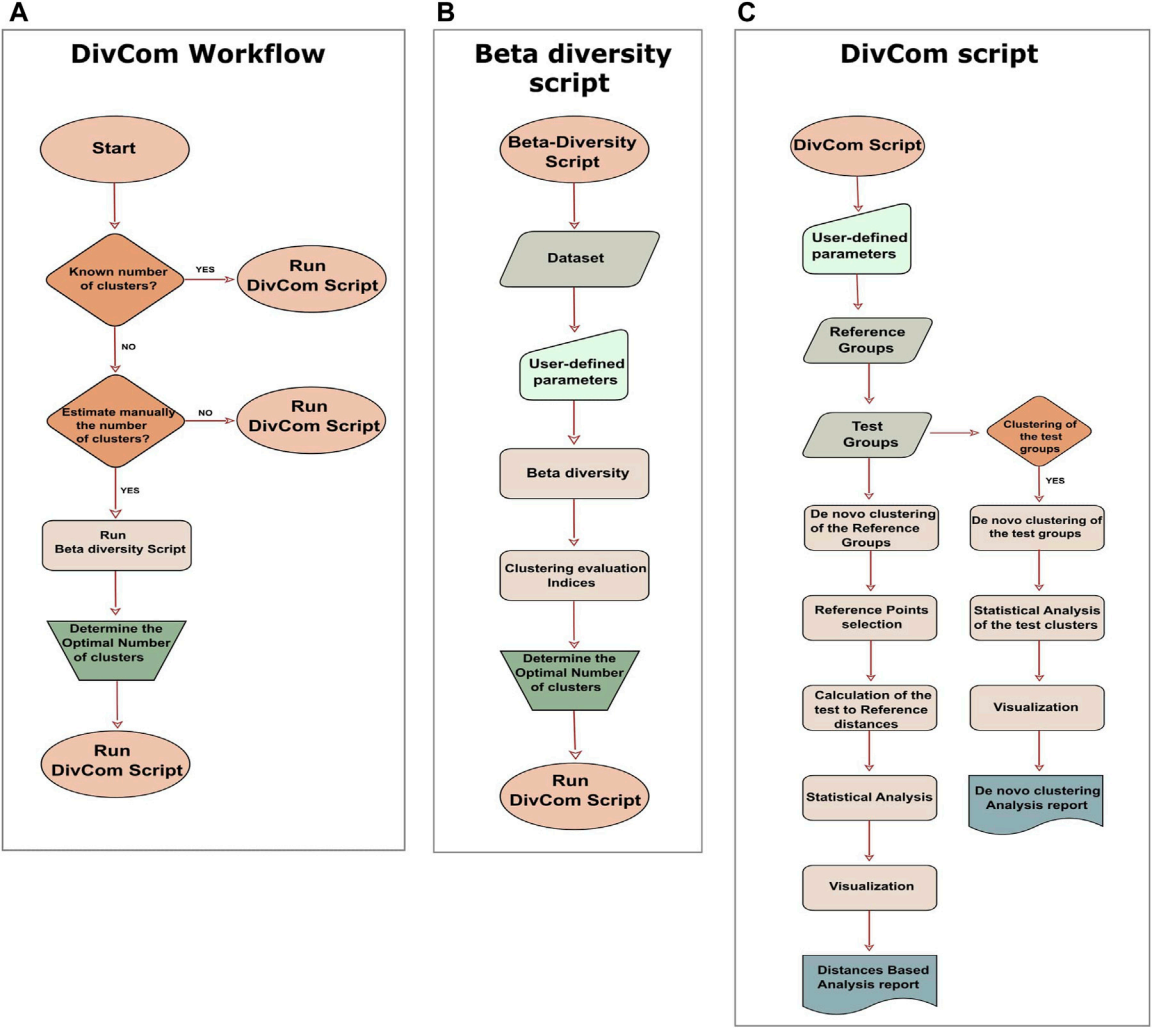
Workflow of the DivCom tool, and the two scripts of the program. **(A)** According to the workflow of the DivCom, the user can execute the beta-diversity to calculate the optimal number of clusters or to directly run the DivCom script. **(B)** The script “Beta-Diversity.R” calculates and visualizes beta diversity between the samples and produces the plots of four different clustering evaluation indices (Calinski-Harabasz, silhouette, prediction strength, and Within Sum of Squares). These outputs provide the user with the necessary information in order to determine the optimal number of clusters for each group. **(C)** The main script is called “DivCom.R” and performs *de novo* clustering to both the reference and test groups, calculates the pairwise distances of the reference and test samples, and finally conducts an automated statistical analysis and produces the final reports. This information contributes to a better understanding of the interrelation between the different groups under study.

DivCom has been implemented in R programming language under version 4.1.2. The tool relies on the functions provided by R packages: ade4, ape, caTools, cluster, cowplot, data.table, dplyr, factoextra, fpc, ggplot2, ggpubr, ggtree, graphics, grid, gridExtra, gtable, GUniFrac, mclust, phangorn, RColorBrewer, stats, tidyr, tools, and vegan. Many of these packages have their own dependencies. In the detailed description of the scripts, some of the key functions are provided, along with the package to which they belong. Also, selected sections of the Rhea pipeline (Lagkouvardos et al., 2017) were modified accordingly and incorporated in the DivCom scripts. The tool consists of two scripts, named “Beta-Diversity.R” and “DivCom.R.” The former is an ancillary script, while the latter is the main script of the tool (Figure 2A).

DivCom is a purely distance-based tool that compares different groups by taking into consideration the phylogenetic distances between observed organisms, and using statistical measures to evaluate the results. Therefore, the Partitioning Around Medoids (PAM) algorithm (Kaufman and Rousseeuw, 2009) is applied to cluster the samples (cluster::pam), and Generalized Unifrac (Chen et al., 2012) is the default distance metric used by the program (GUniFrac::GUniFrac). The statistical hypothesis testing relies on the Wilcoxon rank sum test (Mann and Whitney, 1947; Wilcoxon, 1992) for the continuous variables (stats::wilcox.test), the chi-square test for the categorical variables (stats::chisq.test), permutational analysis of multivariate dispersions (PERMDISP) (Anderson, 2006) for the dispersion similarity comparison of the groups (vegan::betadisper and permutest), and permutational multivariate analysis of variance (PERMANOVA) (Anderson, 2001) for the similarity comparison of the groups (vegan::adonis). All the *p*-values are adjusted using the Benjamini–Hochberg method (Benjamini and Hochberg, 1995) (stats::p.adjust). The multidimensional scaling (MDS) algorithm (Gower, 1966) is applied for the ordination analysis (stats::cmdscale), and finally, scatterplots, boxplots, barplots, and phylograms are used to visualize the findings (ade4::s.class, ggtree, ggtree, ggplot2).

### 2.2 Inputs

The input requirements are minimal as the user has to provide only three mandatory files.

• The first file is an OTU or ASV abundance table which can be either normalized or not. In this table, the rows should represent the OTUs or ASVs, and the columns should represent the samples. In case the table is not normalized, then the first step will be the normalization of the table so the sum of the counts will be equal across all the samples.
• Considering that the generalized Unifrac distance is used as the default distance metric, the second necessary input file is a phylogenetic tree that corresponds to the OTUs or ASVs of the abundance table. If a tree is not available, the user can instead provide a dissimilarity matrix of the samples.
• The final requirement is a mapping file that contains the labels of the samples. The information of the mapping file is necessary for the labeling and assigning of the reference and test groups.

In addition to the files mentioned earlier, the user has to fill out some additional parameters. The desired number of clusters for each group, the name of the reference and test groups, and the type of the produced plots are among these additional requirements. A detailed description for each of these parameters is provided in the scripts and the accompanying documents of the DivCom tool on its GitHub page.

Also, in the initiation phase, the user has to define the names of the input files and then determine which group or groups will serve as the reference dataset. The rest of the samples will be compared with this reference group.

### 2.3 Beta-Diversity Script

Moving on to the actual scripts of the program, the first is named “Beta-Diversity.R” (Figure 2B), and it is a slightly revised version of the “Beta-Diversity” script of the Rhea pipeline. Its purpose is to calculate Beta-Diversity for microbial communities but mainly to provide us with all the necessary information about each group’s optimal number of clusters. The script produces the plots of the Calinski-Harabasz (Caliński and Harabasz, 1974) and the silhouette (Rousseeuw, 1987) index. (fpc::cluster.stats), the Within Sum of Squares (WSS) (factoextra::fviz_nbclust), and the prediction strength (Tibshirani and Walther, 2005) (fpc::prediction. strength) plots and also the plot of the BIC values for six models as they are produced by the model-based clustering based on finite Gaussian mixtures (mclust::Mclust). The purpose of the last plot is to inform us if the dataset consists of a homogenous and uniform distribution so that no substructures exist. If this is true, then the program will propose just one cluster. These measures were selected as they are among the most widely used techniques for clustering validation (Baarsch and Celebi, 2012; Bouveyron and Brunet-Saumard, 2014).

#### 2.3.1 Optimal Number of Clusters

The appraisal of these graphs in conjunction with the prior knowledge of the dataset can help the user decide about the optimal number of clusters for each group. Although we recommend that users make this decision based on their preferences and understandings, among the default outputs of the script a report is included with a recommendation about the optimal number of clusters for each group. To make this suggestion, the script first calculates the optimal number of clusters for each index and then selects the number with the highest frequency. In case of a tie, this suggestion is based on the results of the Calinski-Harabasz index. Even though all indices have their own strengths and weaknesses, we chose to highlight the role of the Calinski-Harabasz index because it is a variance-dependent index that produces higher values when the clusters are compact and well-separated; these characteristics are necessary and highly desirable in our approach. Alternatively, if the user does not wish to evaluate the optimal number of clusters manually, they can omit the Beta-Diversity script and use the integrated option in the main script for automatic calculation of the optimal number of clusters for each group based on the values of the Calinski-Harabasz index. Depending on their preferences, the users can manually evaluate the optimal number of clusters, follow our recommendation, or choose to be automatically calculated by the program (Figure 2A).

### 2.4 DivCom Script

After determining the optimal number of clusters for each group, the user has to run the main script of the tool, which is named “DivCom.R.” DivCom script consists of two main sections (Figure 2C): the first is called “Distances-Based analysis” and the second “*De novo* clustering analysis.” The main difference between them is that in the first part of the analysis, *de novo* clustering is applied only to the reference dataset, while in the second and optional stage, all the groups are clustered, and then compared and analyzed against each other.

#### 2.4.1 Distances-Based Analysis

Proceeding to the actual procedures of the tool, in order to achieve a better insight into the data and take into consideration the unique substructure of the groups, the script performs *de novo* clustering to the samples of the reference group. The PAM algorithm performs this task using the desired number of clusters and the produced distances matrix as inputs. Through this process, the most representative points of the reference group are determined and stored for further use. The medoids of the clusters can be used as the representative points; this is the default and recommended option. Also, the mean or median counts of the OTUs or ASVs can be used as an alternative option to the medoids.

Following the clustering process, the program calculates the distances of the remaining samples to these representative points. Then, each sample is assigned to the closest and probably more relevant to it, in terms of their microbial composition, reference cluster. This procedure results in an indirect clustering of the test samples based only on the distances from the most representative points of the reference group.

Next, a fully automated statistical analysis is conducted. MDS plots visualize the relationship of the reference clusters with their closest test samples. Boxplots present the distances of the samples under study from the nearest reference cluster. Also, tables containing the *p*-values and various statistical measures are printed. Finally, a part of the process is dedicated to analyzing the distribution of the test samples across the clusters of the reference group. This part can assist the user in discovering similar patterns between the reference and test groups.

#### 2.4.2 *De Novo* Clustering Analysis

The second part of the analysis is complementary to the previous section. The main difference is that *de novo* clustering is applied not only to the reference but also to each of the test groups. The user has to specify the desired number of clusters for each test group in the initiation phase. If this information is not provided correctly, then this part of the analysis is omitted.

Assuming that the aforementioned information has been provided, every test group is clustered using the PAM algorithm. Subsequently, every subcluster is compared with the representative points of the reference group. This process results in outputs that compare the structures of the reference and test groups. Therefore, it is easier for the user to reveal the substructural similarities and existing relations between the groups.

Once again, an entirely automated statistical analysis is performed following this procedure. Various descriptive statistics measures for the clusters of the reference and test groups are produced. MDS and boxplots which visualize the relation between the subclusters, and the tables containing *p*-values, distances, and statistical measures are printed. Similar to the previous stage, the distributions of the test samples across the clusters are analyzed and assessed.

Considering that these two sections of the program produce analogous results, the user can compare their outputs and uncover aspects of the dataset that would be difficult to be discovered in any other way.

### 2.5 Outputs

The program produces two detailed reports in the PDF format, one for each of the two steps described earlier. The first file is named “Distances-based report,” and its goal is to present the information about the discrepancy between the reference and the test groups. This report visualizes and statistically investigates the relation of the reference clusters to their closest test samples. The second output is a PDF file called “*De novo* clustering report,” and it aims to present the relationship between the reference and test subclusters. Since *de novo* clustering has been applied to both the control and test groups, this file focuses more on the relationship and the distance-based similarities of the reference clusters with their closest test subclusters.

Each of these reports includes MDS plots and phylograms that illustrate the relationship between the samples. Boxplots present the distances from the selected representative points and tables containing various statistical measures derived from the analysis. In order for the results to be more interpretable by the user, a detailed description is included for each of these elements. Additionally, all the outputs are printed in the PNG or tab format in a separate folder.

### 2.6 Test Dataset

To demonstrate the performance of DivCom and allow users to test the functionality of the tool, a previously unpublished raw sequencing dataset from the iron replacement study of Lee et al. (2017) was used and is publicly available from now on.

This particular dataset was selected as the objective of the study was in line with the requirements of DivCom. This research aimed to assess the effects of Per Oral (PO) and intravenous (IV) iron replacement therapy (IRT) in patients with two types of inflammatory bowel disease (IBD) and a group of non-inflamed (NI) individuals with iron deficiency. The cohort consisted of Crohn’s disease patients (CD, N = 31), ulcerative colitis patients (UC, N = 22), and non-inflamed individuals (NI, N = 19); in total, 62 subjects were involved in this study. The NI individuals were used as the control/reference group, while the CD and UC patients were used as the test groups. All the subjects were randomly separated into PO or IV groups, and they followed the corresponding therapy for 3 months. Therefore, the dataset consisted of two timepoints based on the sampling time; the first timepoint referred to the samples at the baseline (B) and the second to the samples after the 3-month treatment (3M).

The raw sequences were processed through the IMNGS platform (Lagkouvardos et al., 2016), implementing the UNOISE3 (Edgar, 2016) and UPARSE (Edgar, 2013) pipelines, using the default parameters. The number of samples of each category that fulfilled the quality assessment and eventually took part in the final analysis is summarized in Table 1.

**TABLE 1:** Number of samples of each category that were used in the DivCom analysis. In total, 19 samples of the NI group, 26 of the CD group, and 17 of the UC group were selected to participate in the study analysis.

Iron intake	NI (non-inflamed reference group)	CD (Crohn’s disease test group)	UC (ulcerative colitis test group)
PO	9	12	10
IV	10	14	7
Total	19	26	17

## 3 Results

As presented in the introduction, the DivCom approach surpassed the limitations and pitfalls of the currently applied methodology and revealed the true relationship between the groups (Figures 1B and E). Here, using the test dataset of the iron replacement study, we evaluated the performance of our tool in real and complex data, its ability to reproduce parts of the initial analysis, and its contribution to a better understanding of the dataset.

**FIGURE 3:**
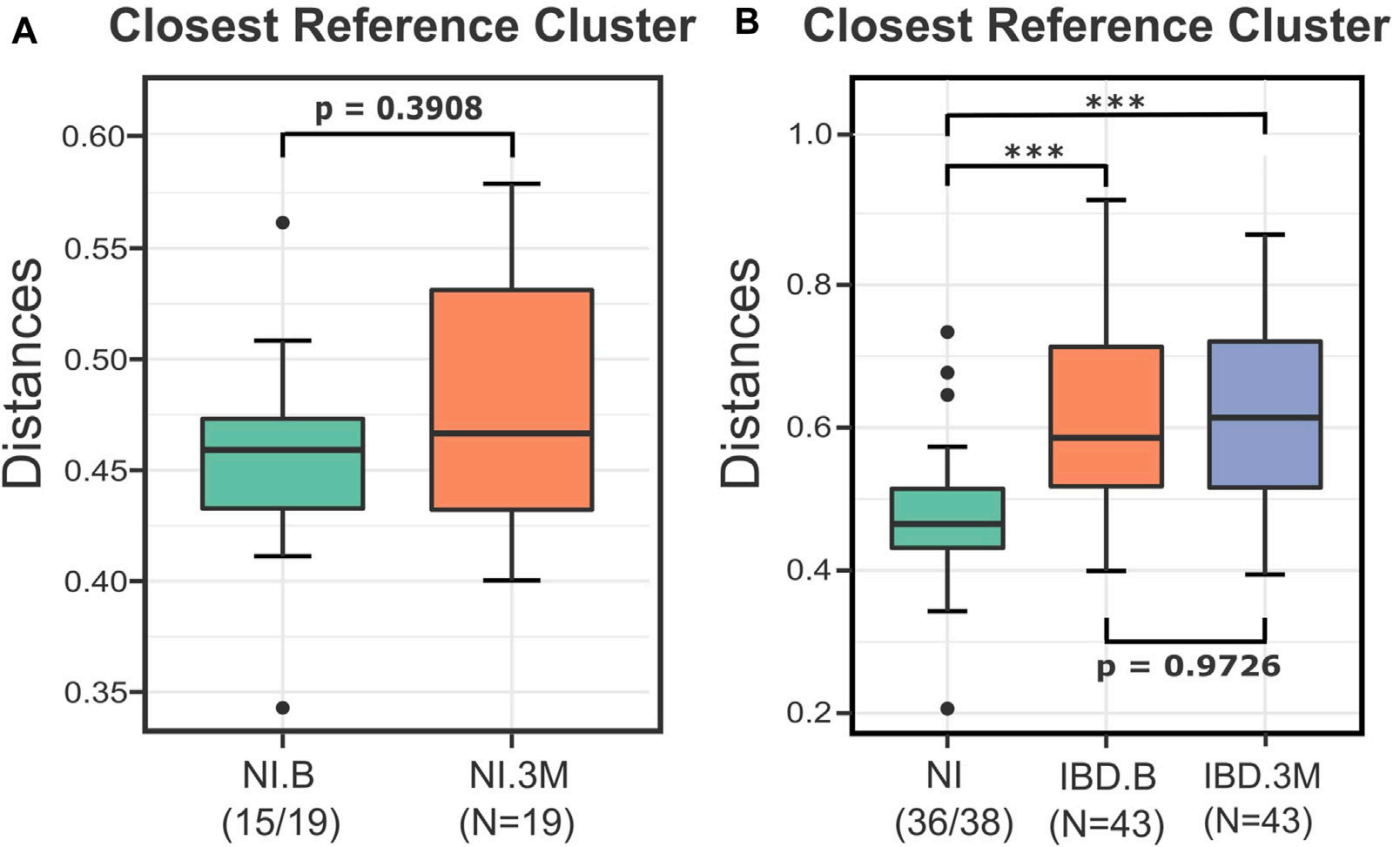
Boxplots presenting the distances of the NI and IBD samples from their closest reference medoids before (B) and after (3M) the treatments. **(A)** The distances of the NI.3M samples from their closest medoid of the NI.B group implied that the iron replacement therapy (IRT) did not affect the microbial composition of NI samples considerably. The two groups were not significantly different (*p* = 0.3908), so for the rest of the DivCom analysis, the samples were merged and used as a unified reference group. **(B)** Boxplots displaying the impact of the iron replacement therapy on the IBD samples. The IRT did not shift the IBD samples closer to the NI group. The distances of the IBD groups (B and 3M) from the reference group of the NI samples are significantly different compared to those of the NI group (*p*<0.001). However, the two groups are highly related to each other (*p* = 0.97). *p*-values: *<0.05; ***<0.01.

In the first step of the analysis, we evaluated the effect of the treatment on the non-inflamed (NI) control samples. As indicated by the Calinski-Harabasz index and verified by the suggestion of the Beta-Diversity script, the pretreatment samples of the NI group (NI.B) were partitioned into four clusters (Supplementary Figure S2A). The distances of all the after-treatment individuals (NI.3M) from these clusters were calculated and then evaluated. These distances indicated that there was no significant difference for the profiles of the non-inflamed (NI) anemic patients before (B) and after (3M) iron treatment (*p* = 0.3908) (Figure 3A). Therefore, since the IRT did not result in consistent changes in the overall microbial profile of the samples, all the NI individuals were merged and used as a unified reference group consisting of 38 profiles. The Calinski-Harabasz index and the recommendation of the Beta-Diversity script supported the existence of two clusters for the entirety of the reference group (Supplementary Figure S2B). Therefore, for the rest of the analysis, the control group of the NI was subdivided into two clusters. The type of treatment (IV, PO) and the sampling time (B, 3M) did not contribute to the formation of these two groups as the chi-square *p*-values were 0.217 for the first case and 0.602 for the second.

Continuing the analysis, we investigated whether the intervention shifted the IBD samples (UC and CD) closer to the NI reference points. The distances of the IBD groups (B and 3M) from the NI reference points were significantly higher than those of the reference group (*p* < 0.001), highlighting in this way the disturbed nature of the IBD profiles. Nevertheless, those distances were not significantly different among time points (IBD.B-IBD.3M) (*p* = 0.96), indicating that the treatment did not affect the median distances of the IBD sample from the NI reference samples (Figure 3B).

**FIGURE 4:**
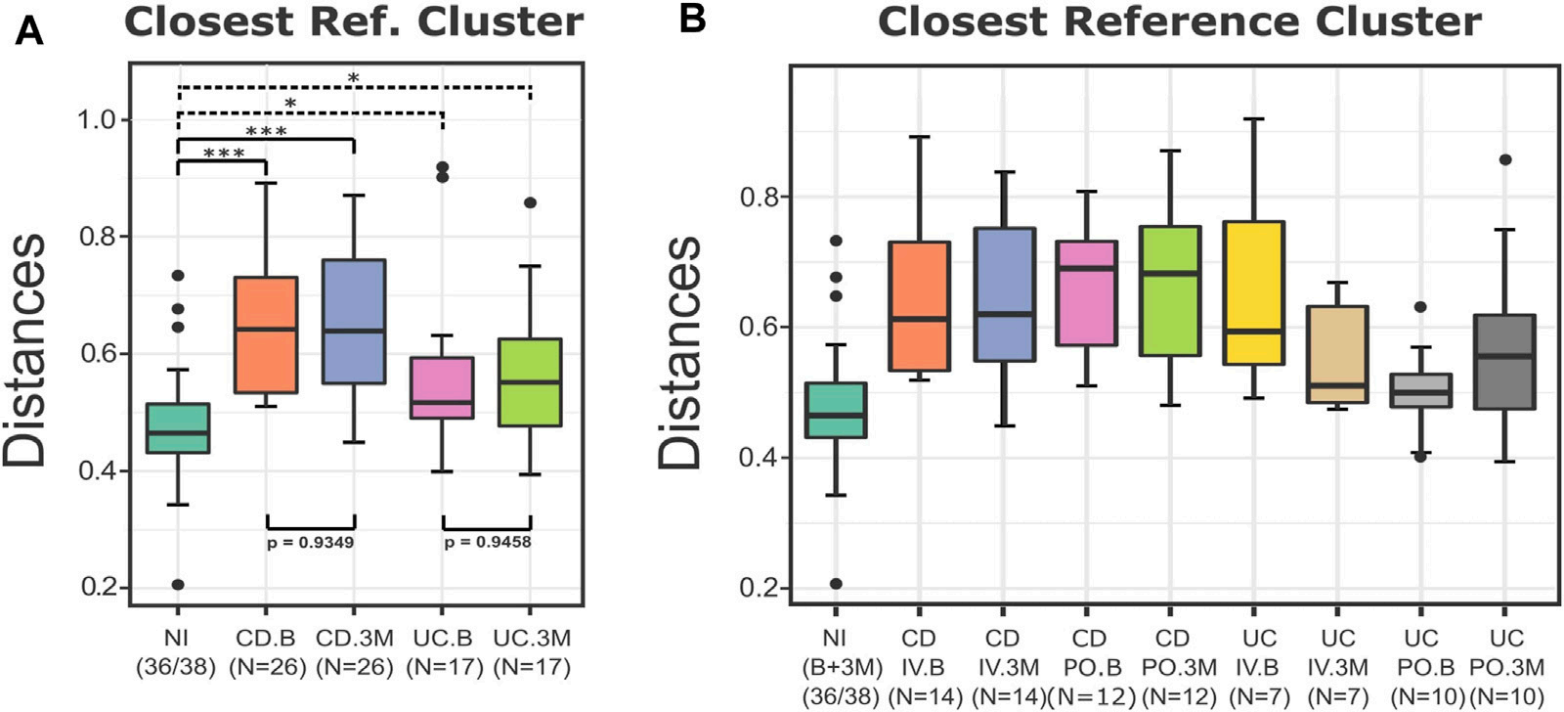
Boxplots of the distances of the test groups from the closest reference medoid of the NI group. **(A)** For the UC and CD groups, the type of the disease and the sampling time did not affect their distances from the NI samples. All the groups were significantly farther from the control group compared to the reference samples (*p* < 0.05). The distances of the CD patients from the control group seem to present overall higher values than the distances of the UC patients from the NI group. **(B)** All the IBD samples were grouped based on the type of the disease, the treatment, and the sampling time. The boxplots of the distances from the closest medoid indicate that the CD groups have a higher level of homogeneity but are farther from the control group compared to the UC groups. On the contrary, most of the UC samples are closer to the NI group, but their distances from the reference group present a higher variance. In particular, the distances of the UC.PO.B group are related to the NI group (*p* = 0.34). *p*-values: *<0.05; ***<0.01.

Next, we repeated the analysis using the sampling time (B or 3M) and the type of disease (CD or UC) as the independent variables. The boxplots of the distances from the closest reference medoid and the statistical testing indicated that the UC and CD groups at the baseline and after the iron replacement were once again significantly farther from the control group of the NI compared to the reference samples (*p* < 0.05) (Figure 4A). Regarding the distances, the CD patients seemed to have a more substantial level of dissimilarity with the NI group than the UC patients. Also, DivCom automatically assigned each IBD profile to its closest NI reference medoid. The integrated chi-square analysis revealed that the samples of the UC group before and after the intervention had similar distribution around the reference medoids to the samples of the NI group (*p* = 0.5786 at the baseline, *p* = 0.2602 at 3 months). On the other hand, this trend was not present for the CD patients (*p* = 0.0070 at the baseline, *p* = 0.0003 at 3 months).

When the IBD samples were labeled based on the disease (CD and UC), the type of the treatment (IV and PO), and the sampling time (B and 3M), it was more evident that the CD groups appeared to have greater distances from the control group than the UC samples (Figure 4B). The IRT seemed to have a more pronounced effect on the UC groups as their samples exhibited a higher variance in terms of their distances from the reference group before and after the treatments. In the UC patients, the type of iron replacement showed trends of differential effect, with the IV group demonstrating a slight decrease and the PO group exhibiting a small increase in the overall distances from the NI. However, in both cases (UC and CD), the distances from the reference group did not change considerably, independent of the type of the iron replacement. On a side note, we revealed that at the baseline, the UC samples chosen to follow the PO treatment seemed to be considerably closer to the NI group than the remaining samples of the UC or CD patients.

**FIGURE 5:**
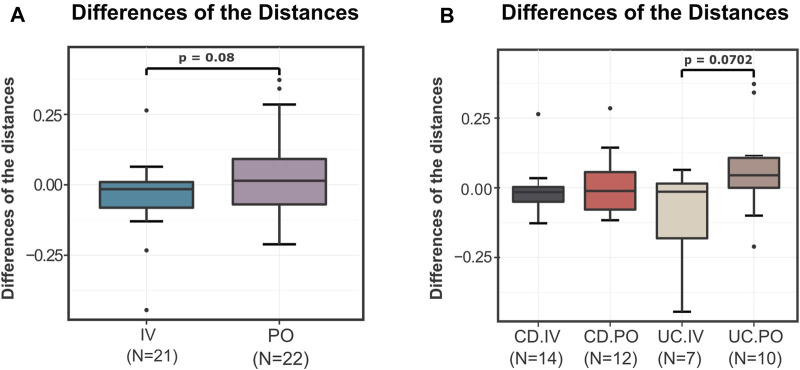
Differences of the “before–after” distances of the test samples from the closest reference medoid of the NI group. **(A)** For the samples of the IV and PO treatments, the differences of the distances from the closest reference medoid before and after the IRT were calculated. The boxplots illustrate the distribution of these differences. The IV treatment seems to have slightly but not significantly better results than the PO treatment (*p* = 0.08). The average differences of the IV samples are negative (-0.0609), while the average differences of the PO treatment are positive (0.09715). **(B)** The IBD samples were compared based on the type of the disease and the treatment. In terms of distance to the reference samples, a differential treatment response is observed on UC patients (*p* = 0.0702). CD patients do not seem to be affected by the mode of treatment, with both resulting in a slight convergence to the reference profiles.

Subsequently, taking advantage of the outputs of the DivCom, a secondary analysis was conducted. The intention was to determine whether the PO or IV treatment had a more profound impact on the distances of the test samples to the reference dataset. Thus, the differences between the distances from the closest medoid after the intervention and those at the baseline were calculated. The average differences of the distances for the IV treatment in both the CD and UC groups were negative (CD = −0.0053, UC = −0.1165). On the other hand, the corresponding differences for the PO treatment in the CD and UC patients were positive (CD = 0.0267, UC = 0.1676). Overall, the statistical comparison of those differences for the two types of treatment showed a trend (*p* = 0.08), indicating that PO treatment led to an increase of distance from the reference samples, while the IV treatment resulted in a decrease (Figure 5A). This difference was mainly due to the differential effect of the treatment type on the UC patients, with the CD patients remaining mostly unaffected (Figure 5B).

**FIGURE 6:**
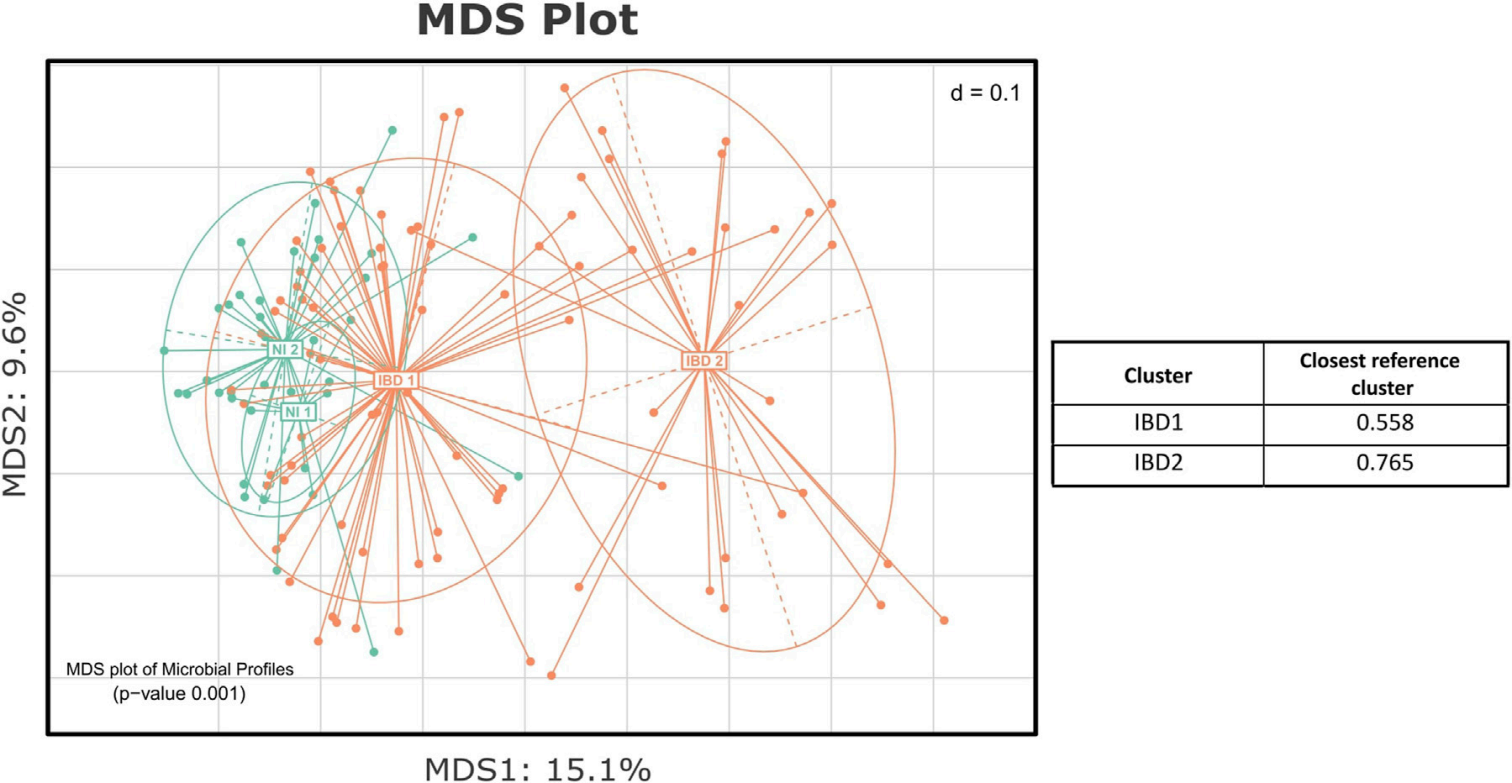
MDS plot presenting the *de novo* clustering of the NI and IBD groups. Both the NI and IBD samples were clustered into two clusters. The IBD 1 subcluster is closer to the reference groups of the NI, and the IBD 2 is considerably more distant. The table presents the median level of dissimilarity of each IBD cluster from its nearest reference cluster.

In order to reveal the test group’s unique substructure, *de novo* clustering was applied to the IBD profiles. As suggested by the Calinski-Harabasz index (Supplementary Figure S2C) and the majority of the other indices as they were produced by the Beta-Diversity script, the IBD group was partitioned into two clusters (Figure 6). One cluster was closer to the NI group, and the other was considerably more distant from the reference samples. This finding was further evaluated through the statistical testing of the corresponding distances of each subcluster from the nearest reference medoid (Supplementary Figure S3). Both the IBD clusters were significantly farther from the NI groups (*p* < 0.05), confirming that the profiles of the patients diverged from those of the control group. Through the automated statistical testing of the DivCom, we verified that the iron therapy did not affect the CD patients. The distribution of the CD samples across the IBD clusters did not change significantly before and after the intervention (*p* = 0.2379). On the contrary, the distribution of UC samples was significantly different after the IRT (*p* < 0.001).

In total, we executed the program five times using the appropriate variables and number of clusters each time (Supplementary Material S1). All the plots and statistical results except Figure 5 were produced directly and automatically by DivCom. The generated outputs underwent only minor editing for complying with the formatting requirements.

## 4 Discussion

### 4.1 DivCom, Iron Dataset, and Beyond

Comparing microbial profiles of different groups can be a challenging process mainly due to the multivariate and multifactorial nature of the data (Ramette, 2007; Paliy and Shankar, 2016). DivCom proposes a new approach for microbial communities’ comparisons that is easily applied using the developed tool. In order to evaluate whether DivCom can produce meaningful results, we employed this methodology to previously produced data that were made public with this work. Next, the basic conclusions of the DivCom analysis are presented and compared with the outcomes of the original publication.

Similar to the results of Lee et al. (2017), we found that the NI group was more homogenous, and the treatment did not considerably affect their overall community composition. Relying on this fact, we treated all the NI samples as a unity during our analysis. In the original article, it was not emphasized that the samples of the UC group were more related to the group of the NI than the group of the CD. In particular, the samples of the UC group chosen to follow the PO treatment were consistently closer to the NI samples. This observation was not mentioned or taken into account in the initial study but was among the default outputs of the DivCom. Sampling imbalances like the above can lead to misleading results when they are not taken into consideration.

In studies exploring the possible differential effect of a treatment on the microbial profiles of two or more groups, the labeling of the subjects is commonly based on their demographics or status characteristics. This process results in dividing the dataset into test and control groups. Traits like age, gender, or disease severity should always be considered and be part of a typical study design in order to avoid biases caused by these factors (Kim et al., 2017; Bharti and Grimm, 2021). However, in addition to the demographic and status characteristics, we argue that the subjects’ baseline microbiome is a significant confounder we should always bear in mind in such analyses. We recommend that an initial screening be performed to map the microbial structure of the cohort, and then subjects be assigned to groups so that the underlying microbial groups are equally represented among the test and control groups. DivCom could assist in this process by revealing the different communities present in the cohorts and creating more balanced experiments.

Also, we verified that although the two treatments overall did not shift the IBD samples significantly closer to the reference group, the IV treatment had slightly better results concerning the distances from the reference medoids. The CD samples seemed to have the same response to the IRT independently of the followed method. In contrast, the UC patients’ microbiomes seemed to be more sensitive to the type of iron replacement, with IV treatment resulting in overall decreased distance from the reference groups and PO negatively affecting the structure of the community reflected in increased distance from the controls. This observation was not accentuated in the original article as the relationship between the treatments and the type of the disease had not been investigated. DivCom can easily highlight such observations through its integral utilization of the distances as the primary method of group comparison.

The *de novo* clustering of the IBD samples showed that one of the two subclusters was close to the reference group, and the other one was farther away. The IRT method did not seem to affect the structure of the IBD clusters. According to chi-square analysis, the after-treatment PO and IV samples were similarly dispersed across the clusters. However, the type of the disease appeared to influence the way the samples are distributed across the IBD clusters. Almost all the UC samples were in the cluster closer to the NI group, while the CD samples were equally dispersed to the two subclusters. Many of these details went unnoticed in the original publication as the structure of the groups had not been taken into account. However, additional variables like the disease severity, age, or diet could also contribute to the observed clustering pattern. In general, *de novo* clustering can provide us with a sense of how well our recorded metadata reflect and explain the grouping of the microbial profiles. The existence of unexpected structures in the dataset could be an indication that factors that had not been predicted or taken into consideration could have a severe impact on the results (Goodrich et al., 2014; Alashwal et al., 2019). Therefore, it is important to always perform this type of analysis in any experiment dealing with microbiome data.

Considering the differences between the original and DivCom analysis, the former was based mainly on the study of dominant bacterial taxa, while the DivCom analysis used beta diversity metrics to summarize the overall community composition. However, both the initial and the current analyses were conducted with respect to the reference group of the NI. Although the samples of the NI group did not have any type of inflammatory bowel disease, the sampling occurred during their hospitalization. Therefore, it would not be appropriate to generalize the results to the wider healthy population. Considering this fact, a universal baseline reference dataset of healthy individuals would be useful for quickly and easily assessing the level of dysbiosis in individual samples (Lloyd-Price et al., 2016; King et al., 2019). If this becomes a reality, then the way will open for more personalized-focused treatments (Zmora et al., 2016; Behrouzi et al., 2019).

### 4.2 Strengths and Limitations of DivCom

Beta diversity is one of the most important parts of the microbiome data analysis; it allows us to explore the relationship between the samples and, by extension, the relation between the different groups under study. As presented and described in the introduction, statistical and structural limitations can produce deceptive outcomes that will consequently affect the rest of the analysis. Most of the time, it is not easy to overcome these obstacles, mainly due to the lack of alternative options. DivCom tries to solve some of these problems by using a distance-based approach that considers the inner structure of the data and reducing the dependency on the results of the statistical tests.

The primary purpose of DivCom is to compare different groups and reveal their interrelation. Therefore, it should be used in studies where two or more groups are compared against each other. The ideal scenario would be when the test groups are compared with control/reference samples. Since the proposed approach uses the pairwise distances of every sample from the reference points, the wrong selection of this dataset may lead to misleading and uninterpretable results. Thus, selecting the reference dataset is an essential part of the process.

An advantage of DivCom is that the sampling size of the dataset and the distribution of the samples across the groups did not considerably affect the overall results. DivCom can produce accurate results independently of the dataset. Although the sampling size does not directly affect the procedure and the outcomes, it is recommended not to use extremely small groups (e.g., 2–3 samples), as in this case, it would be difficult to obtain strong statistical results and draw safe conclusions about the overall trend of the groups.

The *de novo* clustering is a fundamental part of the DivCom methodology. Numerous techniques and algorithms perform unsupervised clustering; among these approaches, model-based clustering methods like Dirichlet multinomial mixtures (DMM) (Holmes et al., 2012) and Dirichlet-tree multinomial mixtures (DTMM) (Bai et al., 2020), density-based clustering algorithms like density-based spatial clustering of applications with noise (DBSCAN) (Ester et al., 1996), or even neural network clustering algorithms like self-organizing tree algorithm (SOTA) (Dopazo and Carazo, 1997) are included. However, here in the DivCom tool, we chose a more conventional approach like the PAM algorithm. PAM does not make any assumptions about the distribution of the samples, can work with any dissimilarity matrix, and forms sphere-like clusters. In particular, the last characteristic is extremely useful as it allowed us to successfully use the concept of the central points as representative points of the clusters. As presented in the introduction, only the use of the medoids/centroids produced results analogous with those we would have obtained if we had performed all the possible calculations and comparisons. All the previously mentioned qualities lead DivCom to be fast and produce accurate and detailed outcomes.

Another benefit of using the DivCom approach is that each sample is studied separately, and the program produces various statistical measures for each of these points. In this way, the user can detect outliers and samples with abnormal behavior more easily, and then further assess them. Identifying and evaluating outliers is not always a straightforward task, so this is an important and maybe overlooked feature of the tool.

DivCom does not require any advanced programming knowledge as the users do not have to edit or modify the code in the scripts; they just need to fill out the required parameters and then execute the program. Each of these parameters is described in the scripts, and clear guidelines are provided so even inexperienced users to be able to use the tool. The workflow of DivCom is flexible and can be personalized depending on the requirements and needs of each user. Also, the results are printed in the form of reports in which each plot and table is explained so it will be easier for the user to interpret the results.

The computational and memory requirements of DivCom can be considered limited. The development and testing of the tool were performed mainly in spec-wise average personal computers (processor: Intel core i5, Ram: 8GB, operating system: Windows 10). The requisite time to complete the process ranged from a few seconds to several minutes, depending on the size of the abundance table. For the dataset used in this study (62 × 254 abundance table), the execution time for the whole analysis was approximately 1 min, while for much larger datasets (e.g., 700 × 5,985 abundance table) the execution time for the whole analysis was no more than 7 min. The only part of the process that has increased computational requirements and is time-consuming is the calculation of the beta diversity (Generalized Unifrac). Therefore, for even larger datasets, it is recommended that the user pre-calculates and provides the matrix of the pairwise distances between the samples in order to speed up the process.

## 5 Conclusion and Future Work

In conclusion, we proposed a novel approach for the analysis of the microbiome datasets. This approach incorporates beta diversity measures used as distance metrics and the technique of *de novo* clustering. This new methodology offers more detailed and well-defined comparisons between different groups under study. An automated tool that applies the suggested method was developed and introduced.

Also, we assessed the performance of DivCom using existing data and comparing the findings with those of the original study. The outcomes showed its effectiveness as we were able to verify some of the key points of the original publication simply by running our tool while discovering and highlighting unnoticed details.

In some cases, the proposed approach outperforms the current methods and techniques that are applied to the beta diversity analysis. Of course, future improvements and optimizations to the tool will render it easier for the user, will simplify the process, and will expand its capability to handle a wider range of possible cases. The use of DivCom combined with the existing tools for downstream microbiome data analysis offers clear advantages and additional information, and therefore should be considered in every microbiome analysis.

## Data Availability

Publicly available datasets were analyzed in this study. These data can be found here: European Nucleotide Archive (https://www.ebi.ac.uk/ena/browser/) with accession number: PRJEB48168.
