# Supplementary material for: DivCom: A Tool for Systematic Partition of Groups of Microbial Profiles Into Intrinsic Subclusters and Distance-Based Subgroup Comparisons
Source: Front Bioinform. 2022 May 12;2:864382. doi: 10.3389/fbinf.2022.864382 (PMC9580884; doi:10.3389/fbinf.2022.864382)
Supplement: Supplementary file 1 [file DataSheet2.PDF]

ANALYSIS WORKFLOW

All the plots presented in the results section are derived directly from the outputs of the DivCom. In total, we run the DivCom script five times using each time different parameters. For the purpose of clarity and reproducibility in the following tables, the input parameters the objective and the output elements for each of these runs are provided.

- **Objective of 1st Run**: Examine if the treatments affected the NI samples.

1st Run	
Variables	***Values***
input_otu:	OTUs-Table.tab
normalized:	NO
tree_or_matrix:	tree
input_tree_or_matrix:	OTUs-NJTree.tre
input_meta:	mapping_file.tab
mapping_column:	Disease_Time
reference_name:	NI.B
reference_clusters:	4
Test_name:	NI.3M
test_clusters:	-
exploratory_columns:	-
central_point:	medoid
plot_type:	Boxplots

**Extracted Elements**		
Element	***Name of the Report***	***Page***
Figure 3A	"Distances Based Report"	6

- **Objective of 2nd Run:** Perform de novo clustering in the IBD group (as a whole) and investigate the relationship between the clusters of the IBD and the NI groups.

**2nd Run**	
**Variables**	**Values**
input_otu:	OTUs-Table.tab
normalized:	NO
tree_or_matrix:	tree
input_tree_or_matrix:	OTUs-NJTree.tre
input_meta:	mapping_file.tab
mapping_column:	Condition
reference_name:	NI
reference_clusters:	2
Test_name:	IBD
test_clusters:	5
exploratory_columns:	Iron_Time, Disease_Time_NI, Treatments,Time
central_point:	medoid
plot_type:	Boxplots

**Extracted Elements**		
Element	***Name of the Report***	***Page***
Figure 6	"De novo clustering report"	3
Figure S3	"De novo clustering report"	12
Chi-square p-values	"De novo clustering report"	7
Chi-square p-values	"De novo clustering report"	9
Chi-square p-values	"Distances Based Report"	15
Chi-square p-values	"Distances Based Report"	17

- **Objective of 3rd Run:** Examine if the treatments shifted the IBD samples closer to the NI groups

3rd Run	
**Variables**	**Values**
input_otu:	OTUs-Table.tab
normalized:	NO
tree_or_matrix:	tree
input_tree_or_matrix:	OTUs-NJTree.tre
input_meta:	mapping_file.tab
mapping_column:	Condition_time
reference_name:	NI
reference_clusters:	2
Test_name:	IBD.B,IBD.3M
test_clusters:	-
exploratory_columns:	-
central_point:	medoid
plot_type:	Boxplots

**Extracted Elements**		
Element	***Name of the Report***	***Page***
Figure 3B	“Distances Based Report”	6

- **Objective of 4th Run:** Explore the relationship between the UC and CD groups with the reference group of the NI.

4th Run	
**Variables**	**Values**
input_otu:	OTUs-Table.tab
normalized:	NO
tree_or_matrix:	tree
input_tree_or_matrix:	OTUs-NJTree.tre
input_meta:	mapping_file.tab
mapping_column:	Disease_Time_NI
reference_name:	NI
reference_clusters:	2
Test_name:	CD.B,CD.3M,UC.B,UC.3M
test_clusters:	-
exploratory_columns:	-
central_point:	medoid
plot type:	Boxplots

Extracted Elements		
Element	***Name of the Report***	***Page***
Figure 4A	“Distances Based Report”	6
Chi-square p-values	“Distances Based Report”	9

- **Objective of 5th Run:** Examine how the type of the disease, the treatment and the time of sampling are connected with the groups of the NI.

5th Run	
**Variables**	**Values**
input_otu:	OTUs-Table.tab
normalized:	NO
tree_or_matrix:	tree
input_tree_or_matrix:	OTUs-NJTree.tre
input_meta:	mapping_file.tab
mapping_column:	Disease_Iron_Time_NI
reference_name:	NI
reference_clusters:	2
Test_name:	CD.IV.B,CD.IV.3M, CD.PO.B,CD.PO.3M, UC.IV.B,UC.IV.3M, UC.PO.B,UC.PO.3M
test_clusters:	-
exploratory_columns:	-
central_point:	medoid
plot_type:	Boxplots

Extracted Elements		
Element	***Name of the Report***	***Page***
Figure 4B	"Distances Based Report"	6
