# Supplementary material for: DivCom: A Tool for Systematic Partition of Groups of Microbial Profiles Into Intrinsic Subclusters and Distance-Based Subgroup Comparisons
Source: Front Bioinform. 2022 May 12;2:864382. doi: 10.3389/fbinf.2022.864382 (PMC9580884; doi:10.3389/fbinf.2022.864382)
Supplement: Supplementary file 2 [file DataSheet1.pdf]

Supplementary Material

1 SUPPLEMENTARY TABLES AND FIGURES

1.1 Figures

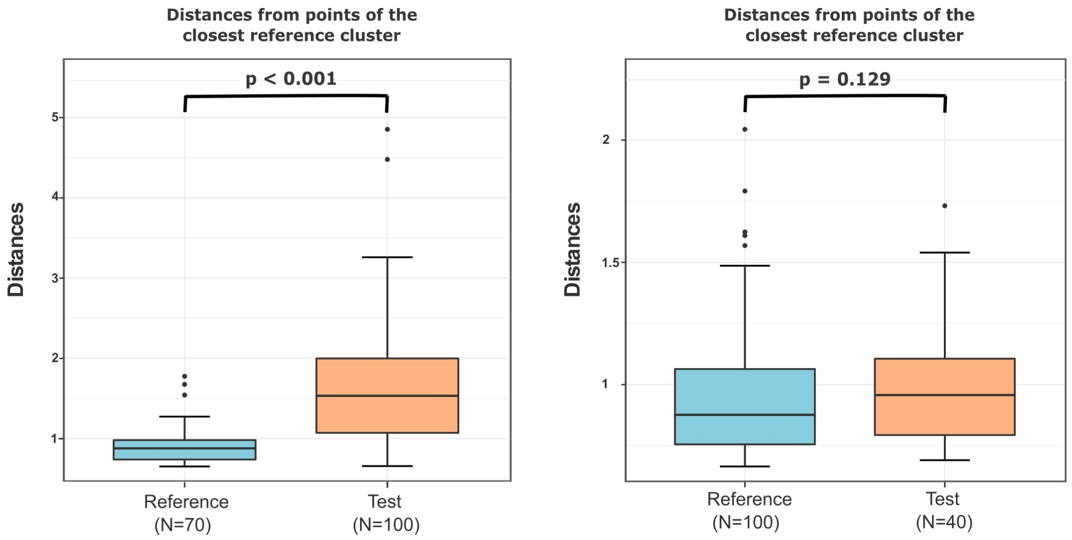

Figure S1. Boxplots presenting the distances of the test samples of Figure 1 from all the reference points of the closest reference cluster. The left panel refers to the dataset of Figure 1A. Similar to Figure 1B, the distances are significantly different (p<0.001), indicating that the two groups are different. The right plot presents the distances of the dataset of Figure 1D; just like the boxplots of Figure 1E, we can infer that the two groups are not significantly different (p=0.129).

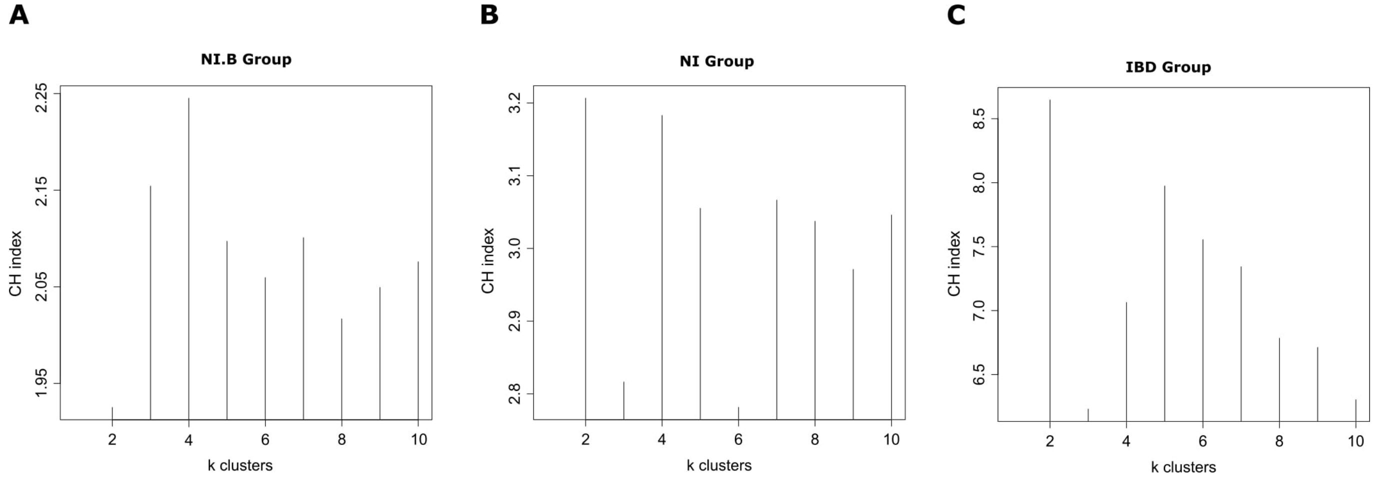

Figure S2. The Calinski-Harabasz (CH) plots for the NI.B, NI, and IBD groups. **A)** According to the CH index, the pretreatment NI group (NI.B) should be clustered into four subgroups. **B)** The CH index plot indicates that the unified group of the NI samples (NI.B+NI.3M) should be clustered into two clusters. **C)** Based on the values of the CH index, the optimal number of clusters for the IBD group is two.

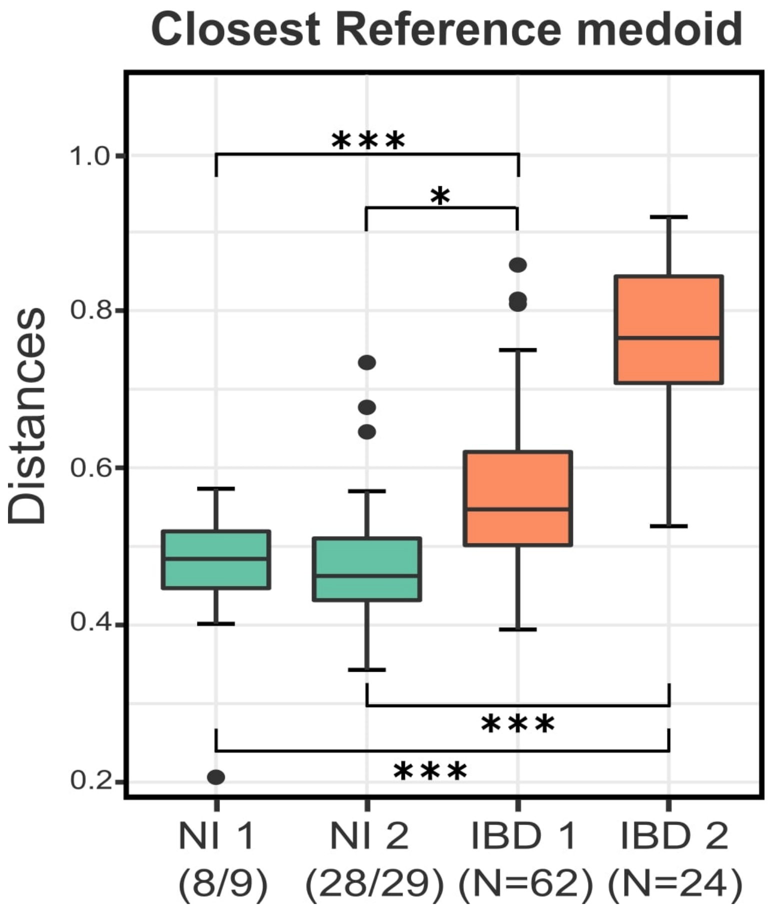

Figure S3. Plot complementary to the MDS Plot of Figure 6 presents the corresponding distances of the IBD samples from the closest reference medoid of the NI group. Similarly to Figure 6, we can observe that one cluster is closer to the NI group, and the other is considerably farther. P-values: *< 0.05; ***< 0.01
